# Event and Comment

**Published:** 1917-12

**Authors:** 


### DENTAL REGISTER

Vol. LXXI DECEMBER, 1917 No. 12

### EVENT AND COMMENT

MEETING OF THE NATIONAL DENTAL ASSOCIATION. The annual meeting this year in New York City was a great success from the standpoint of members in attendance, as nearly 5,000 dentists were enrolled. And considering the size of the city, and the necessarily complicated arrangements for the time and places for holding the various meetings, the entertainment was all that could be expected. The program was full of excellent papers, clinics and exhibits, making a feast of dental matters that satisfied the desires of every one in attendance. Some complaints were registered as to facilities for witnessing and giving clinics. By a peculiar coincidence the western men who were expected to give clinics in oral surgery were barred, because the hospital authorities would not permit them to operate unless they would agree to remain and take care of their patients until they were sufficiently well to leave the hospitals. It seems this is New York law, and consequently there was nothing to be done but to accept the situation and omit all such clinics from the program. The clinics by the Detroit Clinic Club were eagerly attended and seemed to have been the big card of the meeting. They certainly deserved all the praise they received. This may not be the best way to hold clinics in all cases, but it certainly has been the means of developing fine team work and a high order of technical skill. There has been a splendid growth of the Society during the past year and its affairs were never in so good condition as now. Thanks are due to the untiring efforts of the officers. The meeting next year will be held in Chicago.

